# The impact of colorful orthodontic appliances on children’s motivation and treatment perceptions: a single-center cross-sectional study

**DOI:** 10.3389/fdmed.2026.1783018

**Published:** 2026-04-23

**Authors:** Martin Baxmann, Márton Zsoldos, Krisztina Kárpáti

**Affiliations:** Department of Orthodontics and Paediatric Dentistry, Faculty of Dentistry, University of Szeged, Szeged, Hungary

**Keywords:** aesthetic preferences, appliance customization, patient motivation, patient-centered care, pediatric orthodontics, treatment adherence

## Abstract

**Background:**

Color customization of removable orthodontic appliances is widely used in pediatric care, but evidence linking color-related perceptions to motivation and adherence is limited.

**Methods:**

In this retrospective cross-sectional study, routine patient and parent/guardian questionnaires from 316 children aged 8–16 years treated with removable appliances in a private clinic in Germany were analyzed. Outcomes were retrospective patient-reported perceptions of motivation and treatment experience assessed using 5-point Likert items and self-reported adherence (missed a full day of wear in the past week; daily wear-hours). Age group (8–11 vs. 12–16) and gender were key predictors. Likert outcomes were analyzed using Mann–Whitney U tests and adjusted proportional-odds ordinal logistic regression; adherence outcomes were analyzed using chi-square tests and multivariable logistic regression.

**Results:**

Perceptions were favorable: 60.7% agreed/strongly agreed they were motivated to wear the appliance, 53.2% that color made them more eager to wear it, and 94.2% that wear would improve dental health. Younger participants and females reported higher color-related anticipation/enjoyment and lower endorsement that color “does not matter.” Missed full-day wear was reported by 27.3% (70/256); older age was associated with lower odds of missed full-day wear (OR 0.39, 95% CI 0.21–0.72), while color engagement was not. Parent responses were often neutral; younger age was associated with stronger parent endorsement across items.

**Conclusions:**

Color customization is positively perceived and differs by age and gender, but shows limited association with self-reported adherence. Prospective studies incorporating objective wear-time monitoring are warranted.

## 1 Introduction

Orthodontic treatment during childhood and adolescence is central to improving oral function, facial aesthetics, and long-term dental health by correcting malocclusions and guiding craniofacial development (1–3). Early or interceptive treatment can reduce the severity of later orthodontic problems, highlighting the importance of timely care (1). For many pediatric patients, particularly those treated with removable appliances, treatment success depends not only on the clinical plan but also on consistent patient cooperation, including wearing the appliance as prescribed (4).

Patient experience and treatment perceptions are important determinants of engagement. Concerns about discomfort, appearance, and anticipated outcomes can shape satisfaction and willingness to participate in orthodontic therapy (5). Aesthetic features of appliances, including color and design options, may contribute to these perceptions and have been linked to differences in reported motivation and attitudes toward treatment (6, 7). Allowing young patients to select colors may also support autonomy and self-expression, which can be especially relevant during adolescence when peer influence and identity development are heightened (8).

The psychosocial context of orthodontic care is multifactorial. Self-esteem, social confidence, and anxiety can influence how children and adolescents experience visible appliances and respond to treatment demands (9, 10). In addition, preferences and priorities may differ across demographic groups; for example, prior work suggests that aesthetic concerns may be more salient for some female patients, whereas other patients may place greater emphasis on comfort or function (11). Understanding how these factors relate to perceptions of color customization is relevant for patient-centered orthodontic care.

In orthodontics specifically, appliance aesthetics and customization options have been shown to influence patient preferences and perceived acceptability of treatment appliances (7, 12). Beyond orthodontics, customization of visible medical devices (e.g., eyewear and hearing devices) has been associated with improved user acceptance and reduced stigma, supporting the broader rationale for personalization (13–15). However, evidence remains limited regarding how color and customization of removable orthodontic appliances relate to motivation, treatment perceptions, and self-reported adherence in pediatric populations in routine clinical settings.

Therefore, this study explores whether children’s perceptions of colorful, customizable removable orthodontic appliances are associated with self-reported motivation, treatment perceptions, and adherence behaviors among children and adolescents aged 8–16 years. By focusing on routine-care data from a consecutive clinical sample and incorporating both patient- and parent/guardian-reported perceptions alongside adherence indicators, this study helps address a practically relevant but under-researched aspect of pediatric orthodontic care. Differences are evaluated across age and gender groups, and parent-reported perceptions are explored as a secondary outcome. By clarifying these associations, the findings may help inform patient-centered communication and appliance selection in pediatric orthodontic practice.

## 2 Materials and methods

### 2.1 Study design and setting

This retrospective, observational cross-sectional study was conducted at a single private outpatient orthodontic clinic in North Rhine-Westphalia, Germany. Reporting follows the Strengthening the Reporting of Observational Studies in Epidemiology (STROBE) statement.

### 2.2 Participants

All consecutive clinic attendees were included aged 8–16 years who were undergoing treatment with removable orthodontic appliances for any indication (e.g., space maintenance, active correction, retention) during the study period (March–September 2024). Patients were excluded if they were outside the specified age range, were treated exclusively with fixed appliances, or were unable to complete the routine clinical questionnaire due to cognitive or language limitations. For the purposes of this study, ‘treatment with removable orthodontic appliances’ included both active treatment and retention phases when a removable appliance was prescribed.

### 2.3 Data collection

The questionnaire was administered once as part of routine clinical care after completion of the prescribed course of treatment with the removable appliance (including both active and retention phases, as applicable). At this visit, patients and parents/guardians completed the questionnaire reflecting on their treatment experience across the entire period of removable appliance use, perceptions of appliance color, and self-reported adherence during treatment. The questionnaire was not administered before appliance fabrication or at earlier visits; therefore, all variables represent retrospective self-reported perceptions and adherence assessed at a single time point, consistent with a cross-sectional study design. Because the questionnaire was completed after treatment, responses reflect retrospective perceptions of treatment experience and adherence rather than prospectively measured behavior.

### 2.4 Variables and measurement

Primary outcomes were patient-reported motivation and treatment perceptions (individual 5-point Likert items; 1 = strongly disagree to 5 = strongly agree) and self-reported adherence (missed full day in the past week: yes/no; daily wear time in hours/day). Adherence measures were based on patient self-report and therefore reflect perceived wear behavior rather than objectively monitored appliance use. Key independent variables were age group (8–11 vs. 12–16 years) and gender. Age groups were defined *a priori* to reflect dentition and treatment-timing stages commonly referenced in pediatric orthodontics, separating late mixed dentition (approximately 6–12 years) from early permanent dentition (after approximately 12 years), a distinction frequently used when considering interceptive vs. adolescent orthodontic treatment timing (3). Parent/guardian perceptions were evaluated as secondary outcomes (5-point Likert items).

For analyses that required summary measures, we created (a) a patient “color engagement” composite score by averaging three positively framed color items (“look forward,” “more eager,” “more fun”) and a reverse-coded “color does not matter” item (higher values indicate greater color-related engagement), and (b) a parent perception composite score by averaging the three parent/guardian items (willingness, confidence, attitude change). Internal consistency (Cronbach’s *α*) and dimensionality (exploratory factor analysis) were evaluated for these composites.

The questionnaire was developed for routine clinical use in the study clinic to assess patient motivation, perceptions of appliance color, and adherence during treatment with removable orthodontic appliances. The items were designed by the treating orthodontist based on commonly discussed aspects of pediatric orthodontic care, including motivation to wear removable appliances, perceived enjoyment of appliance customization, peer reactions, and expectations regarding dental health improvement. The questionnaire was not derived from a previously validated psychometric instrument; therefore, internal consistency and dimensionality of the composite measures were evaluated statistically as described above. Data were collected as part of routine care; completeness varied by item. The questionnaire was administered in German, the native language of the patient population; the original German-language questionnaire is provided in Supplementary File S1. The questionnaire items were designed to capture clinically relevant aspects of treatment experience commonly discussed during orthodontic care, rather than to serve as a formal psychometric assessment instrument.

### 2.5 Bias and study size

To reduce selection bias, all eligible attendees during the study period were included. Because outcomes were based on routine self-report questionnaires, response and social desirability bias are possible, and parent/guardian items were optional. Because both motivation/perceptions and adherence were self-reported, results may also be influenced by recall bias. The sample size was determined by the number of eligible patients treated during the study period; no formal *a priori* sample size calculation was performed due to the exploratory aim. Given substantial item-level missingness for some variables (notably adherence wear-time and parent/guardian items), the potential for bias due to non-random missingness was evaluated analytically.

### 2.6 Statistical analysis

Analyses were performed in SPSS version 29.0 (IBM Corp., Armonk, NY, USA). Continuous variables are summarized as mean (standard deviation) and categorical variables as *n* (%). Likert-scale outcomes were summarized using response-category distributions (n/%) and, for group comparisons, were analyzed primarily as ordinal outcomes. Between-group comparisons (age group and gender) were conducted using Mann–Whitney U tests (two-sided) as a non-parametric approach and using ordinal logistic regression (proportional-odds cumulative logit models) with age group and gender entered simultaneously to estimate adjusted odds ratios for selecting higher response categories. In these proportional-odds models, the reported odds ratios represent the odds of selecting a higher response category on the Likert scale (greater agreement) relative to lower categories. The proportional-odds assumption was evaluated using the test of parallel lines.

For transparency and comparability with prior literature, independent-samples t-tests were also computed as a supplementary analysis (two-sided), applying Welch’s correction when homogeneity of variance was not met. Assumptions relevant to parametric tests were assessed via Shapiro–Wilk tests of normality (and Q–Q/PP plots) and Levene’s test for homogeneity of variance; multicollinearity was evaluated via tolerance/VIF where applicable.

Categorical outcomes were compared using chi-square tests or Fisher’s exact test as appropriate. Statistical significance was set at *p* < 0.05. Because multiple comparisons were conducted across several exploratory outcomes, statistically significant findings were interpreted cautiously rather than applying formal corrections for multiple testing. Missing data were handled using available-case analysis; for each statistical test, cases with missing (or out-of-range) data for any variable included in that analysis were excluded. Given the exploratory nature of the study and the variable availability of several questionnaire items, available-case analysis was chosen rather than imputation, and results should be interpreted with appropriate caution. Because missingness was substantial for daily wear-hours and parent items, an evaluation of missingness being associated with age group or gender using chi-square tests and with logistic regression models predicting missingness indicators was carried out.

For adherence outcomes, “missed full day” (yes/no) was analyzed using chi-square tests and multivariable binary logistic regression including age group, gender, and the color-engagement composite score. Additional potential determinants of orthodontic adherence (e.g., appliance type, treatment phase, malocclusion severity, and socioeconomic or family-related factors) were not available in the retrospective dataset and therefore could not be included in the regression models. Daily wear-hours (hours/day) were analyzed descriptively and via age/gender group comparisons; additionally, an exploratory multiple linear regression (age group, gender, color engagement) was estimated with bootstrap BCa confidence intervals due to the small subsample and distributional concerns.

### 2.7 Ethics and data protection

This study used anonymized retrospective data derived from routine clinical records and questionnaires. No new data collection, interventions, or patient contact occurred. All data were anonymized prior to analysis. In accordance with Article 9(2)(j) of the General Data Protection Regulation (GDPR) and Section 22 of the German Federal Data Protection Act (BDSG), the processing of anonymized health data for scientific research purposes is permitted without the need for individual informed consent. Because the dataset was fully anonymized prior to analysis and involved retrospective review of routine clinical data, formal Institutional Review Board or ethics committee approval was not required.

## 3 Results

### 3.1 Measurement properties and assumption checks

Shapiro–Wilk tests indicated non-normal distributions for all Likert outcomes across age and gender strata (all *p* < 0.001). Internal consistency was acceptable for the 4-item color engagement scale (Cronbach’s *α*=0.773; *n* = 303) and the 3-item parent perception scale (Cronbach’s *α*=0.840; *n* = 214). These analyses were conducted to assess internal coherence of the composite measures used in the statistical models rather than to establish formal instrument validation. For the color engagement scale, sampling adequacy was acceptable (KMO=0.754) and Bartlett’s test supported factorability [*χ*²(6) = 336.455, *p* < 0.001]; a single factor explained 48.0% of the variance (loadings 0.55–0.80). For the parent perception scale, KMO=0.728 and Bartlett’s *χ*²(3) = 253.967 (*p* < 0.001); a single factor explained 63.6% of the variance (loadings 0.79–0.81). Collinearity diagnostics did not indicate multicollinearity in linear models (tolerance=1.00; VIF=1.00 for age group and gender).

### 3.2 Participants and data completeness

**Figure 1:**
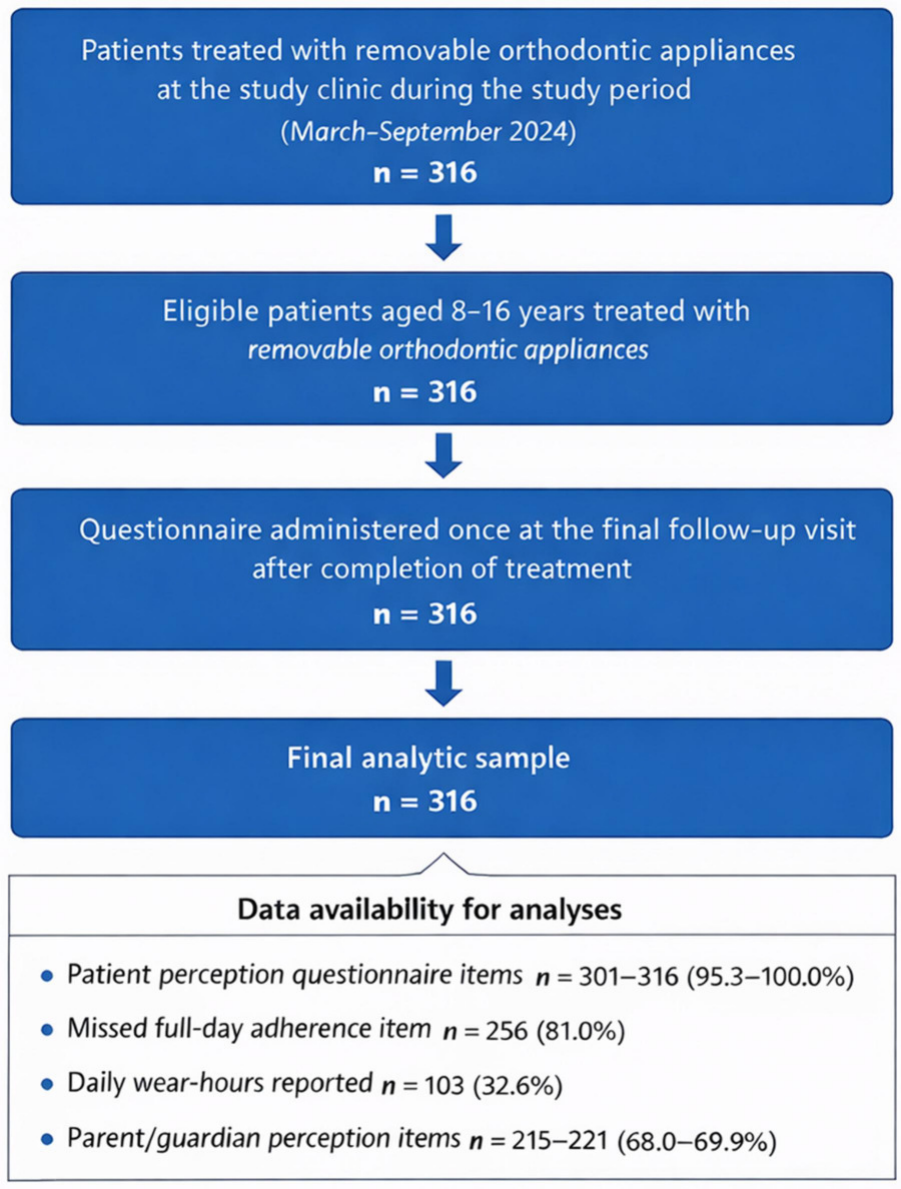
Flow diagram of participant inclusion and data availability.

A total of 316 pediatric patients were included. The process of participant inclusion and questionnaire completion is summarized in Figure 1. Age group was available for 309 patients: 103 (32.6%) were aged 8–11 years and 206 (65.2%) were aged 12–16 years; age group was missing for 7 (2.2%). Gender was available for 315 patients: 173 (54.7%) were female and 142 (44.9%) male; gender was missing for 1 (0.3%). Item-level valid response rates for patient Likert items ranged from 95.3% to 100.0% (Table 1). The missed-full-day adherence item was available for 256/316 (81.0%) participants and daily wear-hours for 103/316 (32.6%). Parent/guardian items were available for 215–221 participants (68.0%–69.9%). In missingness models, parent-item nonresponse was more common in older participants (12–16 vs. 8–11: OR 3.84, 95% CI 2.08–7.11; *p* < 0.001) but was not associated with gender (*p* = 0.665). Wear-hours nonresponse was not associated with age group (*p* = 0.740) but was less common in females than males (OR 0.59, 95% CI 0.36–0.96; *p* = 0.034).

**Table 1:** Patient-reported motivation and perceptions .

Item (patient-reported; 5-point Likert)	Disagree/strongly disagree *n*/*N* (%)	Neutral *n*/*N* (%)	Agree/strongly agree *n*/*N* (%)	Valid *N*
Choosing individual colors for my appliance makes me look forward to receiving it.	18/312 (5.8%)	55/312 (17.6%)	239/312 (76.6%)	312
I feel motivated to wear my orthodontic appliance regularly.	34/313 (10.9%)	89/313 (28.4%)	190/313 (60.7%)	313
The color of my appliance makes me more eager to wear it.	72/316 (22.8%)	76/316 (24.1%)	168/316 (53.2%)	316
Wearing an individually colored orthodontic appliance is more fun than a non-colored one.	68/308 (22.1%)	69/308 (22.4%)	171/308 (55.5%)	308
The color of the appliance does not matter to me.	121/305 (39.7%)	89/305 (29.2%)	95/305 (31.1%)	305
I believe wearing my appliance will improve my dental health.	7/308 (2.3%)	11/308 (3.6%)	290/308 (94.2%)	308
I feel self-conscious about wearing my orthodontic appliance in public.	138/307 (45.0%)	94/307 (30.6%)	75/307 (24.4%)	307
My friends have made positive comments about my appliance.	47/301 (15.6%)	161/301 (53.5%)	93/301 (30.9%)	301

Percentages use the item-specific valid denominator (Valid *N*). “Agree/Strongly agree” combines Likert categories 4–5; “Disagree/Strongly disagree” combines categories 1–2.

Note: All consecutive patients aged 8–16 years treated with removable orthodontic appliances during the study period were eligible. Questionnaires were administered once at the final follow-up visit after completion of treatment. Item-level response availability varied for adherence and parent/guardian perception variables.

### 3.3 Patient-reported motivation and perceptions

As shown in Table 1, most respondents (239/312; 76.6%) agreed or strongly agreed that choosing individual colors made them look forward to receiving their appliance. The majority (190/313; 60.7%) agreed or strongly agreed that they felt motivated to wear their orthodontic appliance regularly. Over half (168/316; 53.2%) agreed or strongly agreed that the color of their appliance made them more eager to wear it, and 171/308 (55.5%) agreed or strongly agreed that wearing an individually colored appliance was more fun than a non-colored one. Most respondents (290/308; 94.2%) agreed or strongly agreed that appliance wear would improve their dental health (Table 1).

### 3.4 Associations with age group and gender

As summarized in Table 2, proportional-odds ordinal logistic regression models were fitted for each patient-reported Likert item, entering age group (8–11 vs. 12–16 years) and gender (female vs. male) simultaneously (Table 2). Compared with older participants (12–16 years), younger participants (8–11 years) had higher odds of stronger agreement that choosing colors made them look forward to receiving the appliance (OR 3.50; *p* < 0.001), that appliance color made them more eager to wear it (OR 1.72; *p* = 0.015), and that a colored appliance was more fun than a non-colored one (OR 2.83; *p* < 0.001). Younger participants had lower odds of endorsing the statement that appliance color does not matter (OR 0.54; *p* = 0.006).

**Table 2:** Associations with age group and gender .

Statement	Model *N*	Age group OR (8–11 vs. 12–16)	*p*-value	Gender OR (Female vs. Male)	*p*-value
Choosing individual colors for my appliance makes me look forward to receiving it.	304	3.50 (2.15–5.70)	<0.001	2.72 (1.73–4.28)	<0.001
I feel motivated to wear my orthodontic appliance regularly.	305	1.08 (0.69–1.68)	0.741	1.36 (0.90–2.08)	0.147
The color of my appliance makes me more eager to wear it.	308	1.72 (1.11–2.65)	0.015	2.07 (1.37–3.13)	<0.001
Wearing an individually colored orthodontic appliance is more fun than a non-colored one.	300	2.83 (1.79–4.48)	<0.001	1.55 (1.02–2.36)	0.039
The color of the appliance does not matter to me.	297	0.54 (0.35–0.84)	0.006	0.55 (0.36–0.83)	0.005
I believe wearing my appliance will improve my dental health.	300	1.12 (0.69–1.81)	0.639	1.45 (0.92–2.28)	0.107
I feel self-conscious about wearing my orthodontic appliance in public.	299	1.20 (0.78–1.85)	0.406	0.81 (0.54–1.21)	0.305
My friends have made positive comments about my appliance.	293	0.87 (0.54–1.38)	0.548	2.22 (1.41–3.48)	<0.001

Odds ratios (OR) are derived from proportional-odds ordinal logistic regression models (logit link). OR > 1 indicates higher odds of selecting a higher response category (greater agreement) on the Likert scale, except where the item is negatively framed (e.g., “color does not matter”). Models include both predictors simultaneously; Model N reflects available-case analysis.

Females had higher odds of stronger agreement that choosing colors made them look forward to receiving the appliance (OR 2.72; *p* < 0.001), that color made them more eager to wear it (OR 2.07; *p* < 0.001), that a colored appliance was more fun (OR 1.55; *p* = 0.039), and that friends had made positive comments (OR 2.22; *p* < 0.001). Females had lower odds of endorsing the statement that appliance color does not matter (OR 0.55; *p* = 0.005).

No statistically significant adjusted associations were observed for feeling motivated to wear the appliance regularly, belief that appliance wear would improve dental health, or self-consciousness about wearing the appliance in public (all *p* > 0.05; Table 2). Mann–Whitney U sensitivity analyses showed the same overall pattern of significant vs. non-significant findings.

### 3.5 Adherence outcomes

Among participants with available missed-full-day adherence data (*n* = 256), 70 (27.3%) reported missing at least one full day in the past week and 186 (72.7%) reported no missed full day. Missed full-day wear was reported more frequently in the 8–11-year group than the 12–16-year group [32/81 (39.5%) vs. 38/169 (22.5%); *χ*²(1) = 7.87; *p* = 0.005] and more frequently in males than females [38/113 (33.6%) vs. 32/142 (22.5%); *χ*²(1) = 3.89; *p* = 0.049].

As shown in Table 3, in multivariable logistic regression including age group, gender, and the color-engagement composite score (*n* = 249, listwise), older age (12–16 vs. 8–11) was associated with lower odds of reporting a missed full day (OR 0.39; 95% CI 0.21–0.72; *p* = 0.002). Gender (female vs. male: OR 0.59; 95% CI 0.33–1.06; *p* = 0.077) and the color-engagement score (per 1-point increase: OR 0.80; 95% CI 0.55–1.17; *p* = 0.250) were not statistically significant predictors.

**Table 3:** Adherence outcomes.

Outcome/model	Predictor	Effect estimate (95% CI)	*p*-value
Missed full day (Yes) – logistic regression (*n* = 249)	Age group (12–16 vs. 8–11)	OR 0.39 (0.21–0.72)	0.002
Missed full day (Yes) – logistic regression (*n* = 249)	Gender (Female vs. Male)	OR 0.59 (0.33–1.06)	0.077
Missed full day (Yes) – logistic regression (*n* = 249)	Color-engagement score (per 1-point)	OR 0.80 (0.55–1.17)	0.250
Daily wear-hours – bootstrapped linear regression (*n* = 99)	Age group (older vs. younger)	B 3.27 (0.70–5.81)	0.016
Daily wear-hours – bootstrapped linear regression (*n* = 99)	Gender (Female vs. Male)	B 0.76 (−2.13–3.46)	0.607
Daily wear-hours – bootstrapped linear regression (*n* = 99)	Color-engagement score (per 1-point)	B −0.13 (−1.97–1.47)	0.880

Missed full day coded Yes/No was analyzed using binary logistic regression; effect estimates are reported as odds ratios (OR).

Daily wear-hours were reported by 103/316 (32.6%) participants (mean 14.2 h/day, SD 6.8, range 0–24). Wear-hours did not differ by age group (Mann–Whitney *p* = 0.053; *n* = 100) or gender (Mann–Whitney *p* = 0.368; *n* = 102). In an exploratory bootstrapped multiple linear regression predicting wear-hours (age group, gender, and color-engagement score; *n* = 99), the overall model explained a small proportion of variance [R² 0.058; F(3,95) = 1.94; *p* = 0.129]. The age-group coefficient was positive (B = 3.27 h/day; BCa 95% CI 0.70–5.81; *p* = 0.016), while gender (*p* = 0.607) and color engagement (*p* = 0.880) were not statistically significant. In sensitivity analyses comparing patient Likert outcomes by missed full-day status, no item-level differences reached statistical significance (all *p* ≥ 0.05).

### 3.6 Parent/guardian-reported perceptions

Among parents/guardians with valid responses, 83/221 (37.6%) agreed or strongly agreed that appliance color influences the child’s willingness to wear the appliance, 73/220 (33.2%) agreed or strongly agreed that the child feels more confident wearing a colored appliance, and 53/215 (24.7%) agreed or strongly agreed that the child’s attitude toward orthodontic treatment improved after receiving a colored appliance. Neutral responses were common across items (38.9%, 36.8%, and 45.6%, respectively). As shown in Table 4, parent response distributions and adjusted associations are presented.

**Table 4:** Parent/guardian-reported perceptions.

Parent/guardian statement	Disagree/strongly disagree *n*/*N* (%)	Neutral *n*/*N* (%)	Agree/strongly agree *n*/*N* (%)	Age OR (8–11 vs. 12–16) (95% CI); *p*	Gender OR (Female vs. Male) (95% CI); *p*
Color influences my child’s willingness to wear the appliance.	52/221 (23.5%)	86/221 (38.9%)	83/221 (37.6%)	1.81 (1.10–2.97); 0.020	2.12 (1.29–3.48); 0.003
My child feels more confident wearing a colored appliance.	66/220 (30.0%)	81/220 (36.8%)	73/220 (33.2%)	1.98 (1.21–3.25); 0.007	1.07 (0.66–1.73); 0.796
I noticed a change in my child’s attitude toward orthodontic treatment after receiving the colored appliance.	64/215 (29.8%)	98/215 (45.6%)	53/215 (24.7%)	1.90 (1.14–3.18); 0.014	1.62 (0.98–2.69); 0.059

Percentages use the item-specific valid denominator (*N*). Odds ratios (OR) are from proportional-odds ordinal logistic regression models including age group and gender simultaneously; OR > 1 indicates higher odds of endorsing higher response categories (greater agreement).

In adjusted proportional-odds ordinal logistic regression models including age group and gender simultaneously, younger age (8–11 vs. 12–16 years) was associated with stronger endorsement of all three parent-reported items (ORs 1.81–1.98, *p*-values 0.020, 0.007, and 0.014). Female gender (vs. male) was associated with stronger endorsement for the willingness item (OR 2.12; *p* = 0.003), was not associated with the confidence item (*p* = 0.796), and showed a borderline association for attitude change (OR 1.62; *p* = 0.059). Tests of the proportional-odds (parallel lines) assumption were not statistically significant for these models (*p* = 0.231–0.926). For the parent perception composite score, Mann–Whitney U tests indicated higher scores in the 8–11-year group (U = 4,495.5; *p* = 0.004; r = 0.19) and in females (U = 5,182.0; *p* = 0.049; r = 0.13).

## 4 Discussion

Using routinely collected clinical data through a retrospective cross sectional design, this study provides a pragmatic assessment of children and adolescents treated with removable orthodontic appliances and found generally favorable perceptions of color customization and high self-reported motivation. This work extends the limited evidence base on removable-appliance color customization by examining perception data alongside adherence indicators and including parent/guardian perspectives from a real-world clinical setting. Higher ratings for anticipation, eagerness, and enjoyment related to colored appliances were observed in younger participants and in females, while several other perceptions (e.g., dental health benefit) were consistently positive across subgroups. These findings support the clinical relevance of aesthetic considerations in pediatric orthodontics and are consistent with prior research indicating that appearance, material properties, and personalization can influence appliance preferences and treatment experience (6, 7, 16).

Personalization may support engagement by increasing perceived autonomy and making an appliance feel more acceptable in social settings, particularly in developmental stages where peer context and identity are salient (15, 17). Similar themes have been reported for other visible health devices, where design and customization options have been linked to user acceptance and reduced stigma (13–15). The observed subgroup differences were generally small-to-moderate in magnitude, suggesting meaningful but not uniform variation in preferences.

Age-related findings warrant nuanced interpretation. Younger participants reported stronger positive perceptions toward color selection, yet missed full-day wear was reported more often in this group. In adjusted adherence models, age group remained associated with missed full-day wear, whereas the color-engagement composite score was not. This pattern suggests that enthusiasm for customization does not necessarily translate into consistent adherence and that practical factors (caregiver involvement, routines, school schedules, and comfort) likely play important roles alongside motivation. Among the subset with wear-hours data, older participants tended to report longer daily wear time in exploratory analyses, although wear-time conclusions remain tentative given substantial missingness.

Gender differences were also modest but consistent across multiple items, with females reporting higher anticipation, eagerness, enjoyment, and more positive peer feedback. Prior work has described gender-related differences in orthodontic service uptake and appliance perceptions (11, 18). These results should be interpreted as group-level patterns rather than deterministic differences, as individual preferences vary widely.

Associations with adherence were limited. Missed full-day wear showed minimal relationships with most motivation and color-perception items; the clearest difference involved perceived peer feedback, with lower positive comments reported among those who missed wear. Wear-time data were incomplete, and objective adherence measures were not available. Taken together, these findings suggest that appliance aesthetics may contribute to treatment experience but represent only one of several factors influencing adherence behavior. Importantly, the absence of an association between the color-engagement composite and adherence outcomes in adjusted models suggests that positive color-related perceptions alone may not overcome adherence barriers.

Parent/guardian responses showed substantial neutral ratings and mixed endorsement that color influences willingness, confidence, or attitude toward treatment. However, adjusted ordinal models indicated higher parent endorsement of color-related willingness, confidence, and attitude change for younger children, with a more limited gender association (primarily for willingness). This may reflect variability in how visible day-to-day wear behaviors are to caregivers, differences in child–parent communication, or heterogeneity in family expectations. Because parent-item missingness was higher in older participants, age-related parent findings should be interpreted cautiously.

Several limitations should be considered. The single-center retrospective design limits generalizability and prevents causal inference. Because the questionnaire was administered after treatment completion, responses reflect retrospective perceptions of treatment experience and adherence and may therefore be influenced by recall bias. Outcomes were based on routine self-reported (and parent-reported) questionnaire items rather than objective behavioral measures, and self-reported adherence indicators—including missed full days of wear and reported daily wear hours—may be affected by recall and social desirability bias and may not fully reflect actual appliance use. Several variables had substantial missing data, particularly daily wear-time and parent-reported items, and analyses were therefore conducted using available-case data; although patterns of missingness were explored analytically, the potential for bias due to non-random missingness cannot be excluded.

In addition, the retrospective dataset did not contain several known determinants of orthodontic adherence, including appliance type, treatment phase, baseline malocclusion severity, socioeconomic context, parental supervision, and other psychosocial factors, and the observed associations may therefore be influenced by unmeasured confounding. The questionnaire was developed for routine clinical use and was not a formally validated psychometric instrument; although internal consistency and exploratory factor structure supported the constructed composite measures, full validation and test–retest reliability could not be assessed within the cross-sectional dataset. However, the use of a retrospective design and a clinic-developed questionnaire reflects the pragmatic aim of capturing patient-reported perceptions within a real-world clinical setting, where standardized psychometric instruments are not routinely implemented. This approach allowed inclusion of a consecutive, practice-based sample and provided insight into patient experiences as they occur in routine care, rather than under controlled research conditions. Accordingly, the findings should be interpreted as exploratory and hypothesis-generating, providing a basis for future studies using validated instruments and prospective designs.

Finally, because multiple subgroup comparisons were performed, statistically significant findings should be interpreted cautiously and considered exploratory. Despite these limitations, the study also has strengths, including the consecutive clinical sample and the inclusion of both patient and parent perspectives in a real-world clinical setting, and the use of ordinal regression models and non-parametric sensitivity analyses reduces dependence on distributional assumptions when analyzing Likert-scale outcomes.

Clinically, offering color options may represent a low-burden patient-centered approach to support acceptability and engagement, particularly for patients who value aesthetic customization. For younger patients, customization may be most effective when paired with structured adherence support (caregiver involvement, reminders, and proactive management of discomfort). Given that adherence outcomes were more strongly associated with demographic factors than with color-engagement perceptions, adherence support strategies should remain central regardless of aesthetic customization. Future studies should use prospective, multicenter designs, validated instruments, and objective wear-time monitoring to clarify the relationship between personalization and adherence over time.

Overall, color customization of removable orthodontic appliances was associated with more positive motivation-related perceptions, particularly among younger patients and females, whereas associations with self-reported adherence were modest and parent/guardian ratings were frequently neutral. These findings suggest that offering color options may be associated with more positive treatment perceptions, while also indicating that customization alone is unlikely to address adherence barriers and should be paired with established support strategies (e.g., caregiver involvement, reminders, and proactive management of discomfort). Given the retrospective cross-sectional design, reliance on self-report, and item-level missingness, causal inferences cannot be drawn. Prospective multicenter studies incorporating objective wear-time monitoring (e.g., electronic sensors) and validated psychosocial assessments are warranted to determine whether aesthetic personalization translates into sustained adherence and improved clinical outcomes.

## Data Availability

The datasets presented in this study can be found in online repositories. The names of the repository/repositories and accession number(s) can be found below: https://zenodo.org/doi/10.5281/zenodo.11372659.
