# Supplementary material for: The impact of colorful orthodontic appliances on children’s motivation and treatment perceptions: a single-center cross-sectional study
Source: Front Dent Med. 2026 Apr 23;7:1783018. doi: 10.3389/fdmed.2026.1783018 (PMC13149403; doi:10.3389/fdmed.2026.1783018)
Supplement: Supplementary file 1 [file Datasheet1.pdf]

Fragebogen zur Studie über kieferorthopädische Apparaturen

Patientenidentifikationsnummer:

Informationen über Teilnehmer

1. Alter:
   ☐ 8-11 Jahre
   ☐ 12-16 Jahre

2. Geschlecht:
   ☐ Weiblich, identifiziert sich als hauptsächlich weiblich
   ☐ Männlich, identifiziert sich als hauptsächlich männlich
   ☐ Möchte ich nicht sagen

Motivation und Wahrnehmung

(Alle Antworten auf einer Likert-Skala von 1 = stimme überhaupt nicht zu bis 5 = stimme voll und ganz zu)

3. Wenn ich individuelle Farben für mein Gerät wähle, freue ich mich darauf, es zu erhalten
   ☐ 1 = Stimme überhaupt nicht zu
   ☐ 2 = Stimme nicht zu
   ☐ 3 = Stimme weder zu noch nicht zu
   ☐ 4 = Stimme zu
   ☐ 5 = Stimme voll und ganz zu

4. Ich fühle mich motiviert, meine kieferorthopädische Apparatur regelmäßig zu tragen.
   ☐ 1 = Stimme überhaupt nicht zu
   ☐ 2 = Stimme nicht zu
   ☐ 3 = Stimme weder zu noch nicht zu
   ☐ 4 = Stimme zu
   ☐ 5 = Stimme voll und ganz zu

5. Die Farbe meiner Zahnspange macht, dass ich mehr Lust habe, sie zu tragen.
   ☐ 1 = Stimme überhaupt nicht zu
   ☐ 2 = Stimme nicht zu
   ☐ 3 = Stimme weder zu noch nicht zu
   ☐ 4 = Stimme zu
   ☐ 5 = Stimme voll und ganz zu

6. Ich habe das Gefühl, dass das Tragen einer individuell gefärbten Zahnspange mehr Spaß macht als das Tragen einer farblosen Apparatur.
   - ☐ 1 = Stimme überhaupt nicht zu
   - ☐ 2 = Stimme nicht zu
   - ☐ 3 = Stimme weder zu noch nicht zu
   - ☐ 4 = Stimme zu
   - ☐ 5 = Stimme voll und ganz zu

7. Ich denke, die Farbe des Geräts ist für mich nicht wichtig.
   - ☐ 1 = Stimme überhaupt nicht zu
   - ☐ 2 = Stimme nicht zu
   - ☐ 3 = Stimme weder zu noch nicht zu
   - ☐ 4 = Stimme zu
   - ☐ 5 = Stimme voll und ganz zu

8. Ich glaube, dass das Tragen meiner Zahnspange meine Zahngesundheit verbessern wird.
   - ☐ 1 = Stimme überhaupt nicht zu
   - ☐ 2 = Stimme nicht zu
   - ☐ 3 = Stimme weder zu noch nicht zu
   - ☐ 4 = Stimme zu
   - ☐ 5 = Stimme voll und ganz zu

9. Es ist mir unangenehm, meine kieferorthopädische Apparatur in der Öffentlichkeit zu tragen.
   - ☐ 1 = Stimme überhaupt nicht zu
   - ☐ 2 = Stimme nicht zu
   - ☐ 3 = Stimme weder zu noch nicht zu
   - ☐ 4 = Stimme zu
   - ☐ 5 = Stimme voll und ganz zu

10. Meine Freunde haben sich positiv über mein Gerät geäußert.
   - ☐ 1 = Stimme überhaupt nicht zu
   - ☐ 2 = Stimme nicht zu
   - ☐ 3 = Stimme weder zu noch nicht zu
   - ☐ 4 = Stimme zu
   - ☐ 5 = Stimme voll und ganz zu

Überprüfung der Einhaltung

11. Wie viele Stunden pro Tag trägst Du Deine Zahnspange? (Offene Frage)

12. Hast Du Deine Zahnspange in der letzten Woche einen ganzen Tag lang nicht getragen?
    ☐ Ja
    ☐ Nein

Die Wahrnehmung der Eltern/Erziehungsberechtigten
(Alle Antworten auf einer Likert-Skala von 1 = stimme überhaupt nicht zu bis 5 = stimme voll und ganz zu)

13. Ich glaube, dass die individuell gewählte Farbe der Zahnspange die Bereitschaft meines Kindes beeinflusst, sie zu tragen.
    ☐ 1 = Stimme überhaupt nicht zu
    ☐ 2 = Stimme nicht zu
    ☐ 3 = Stimme weder zu noch nicht zu
    ☐ 4 = Stimme zu
    ☐ 5 = Stimme voll und ganz zu

14. Ich glaube, mein Kind fühlt sich selbstbewusster, wenn es eine farbige Apparatur trägt.
    ☐ 1 = Stimme überhaupt nicht zu
    ☐ 2 = Stimme nicht zu
    ☐ 3 = Stimme weder zu noch nicht zu
    ☐ 4 = Stimme zu
    ☐ 5 = Stimme voll und ganz zu

15. Ich habe festgestellt, dass sich die Einstellung meines Kindes zur kieferorthopädischen Behandlung geändert hat, nachdem es individuelle Farben für die Apparatur ausgewählt hatte.
    ☐ 1 = Stimme überhaupt nicht zu
    ☐ 2 = Stimme nicht zu
    ☐ 3 = Stimme weder zu noch nicht zu
    ☐ 4 = Stimme zu
    ☐ 5 = Stimme voll und ganz zu

Zusätzliche Kommentare

16. Bitte teilen Sie uns weitere Gedanken oder Erfahrungen mit, die Sie in Bezug auf Ihre kieferorthopädische Apparatur haben. (Offen)
